# Unravelling structural ambiguities in lithium- and manganese-rich transition metal oxides

**DOI:** 10.1038/ncomms9711

**Published:** 2015-10-29

**Authors:** Alpesh Khushalchand Shukla, Quentin M. Ramasse, Colin Ophus, Hugues Duncan, Fredrik Hage, Guoying Chen

**Affiliations:** 1 Energy Storage and Distributed Resources Division, Lawrence Berkeley National Laboratory, Berkeley, California 94720, USA; 2 SuperSTEM Laboratory, SciTech Daresbury Campus, Daresbury WA4 4AD, UK; 3 National Center for Electron Microscopy, Molecular Foundry, Lawrence Berkeley National Laboratory, Berkeley, California 94720, USA

## Abstract

Although Li- and Mn-rich transition metal oxides have been extensively studied as high-capacity cathode materials for Li-ion batteries, the crystal structure of these materials in their pristine state is not yet fully understood. Here we apply complementary electron microscopy and spectroscopy techniques at multi-length scale on well-formed Li_1.2_(Ni_0.13_Mn_0.54_Co_0.13_)O_2_ crystals with two different morphologies as well as two commercially available materials with similar compositions, and unambiguously describe the structural make-up of these samples. Systematically observing the entire primary particles along multiple zone axes reveals that they are consistently made up of a single phase, save for rare localized defects and a thin surface layer on certain crystallographic facets. More specifically, we show the bulk of the oxides can be described as an aperiodic crystal consisting of randomly stacked domains that correspond to three variants of monoclinic structure, while the surface is composed of a Co- and/or Ni-rich spinel with antisite defects.

## Abstract (web summary)

Lithium and manganese-rich transition metal oxides are a class of promising battery electrodes but their structures are a subject of a controversial debate. Here, the authors use a variety of materials characterization tools to unravel the structural ambiguities in these materials.

## Graphical abstract

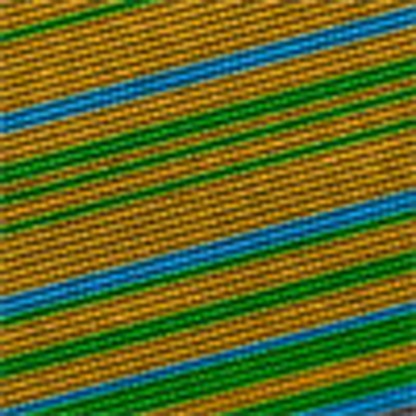

Layered transition metal oxides (LiMO_2_, where M is usually Co, Mn, Ni or some combination thereof) are presently used as cathode materials for secondary lithium-ion batteries in consumer electronics, as they provide reversible capacities from about 140 to 190 mAh g^−1^. It is known that increasing the lithium and manganese contents in these materials to a general composition of Li_1+*x*_M_1−*x*_O_2_ can lead to much higher capacities, typically >250 mAh g^−1^ (ref. 1). Although this class of Li- and Mn-rich transition metal oxides (referred to as LMRTMOs hereafter) was introduced nearly a decade ago, their commercial application has been hindered by severe shortcomings in the materials, including a large first-cycle irreversible capacity loss^2^, voltage and capacity fade^3^, d.c. resistance rise at a low state of charge and transition metal dissolution^4^. Studies have attributed these issues to the structural changes occurring during first-cycle activation and prolonged cycling, yet the crystal structure of the pristine oxides is still a matter of debate. Much of the research efforts have been focused on electrochemistry^5^,^6^,^7^,^8^,^9^ and phase transformation^10^,^11^,^12^,^13^ studies, and several attempts that have been made to solve the crystal structure of the pristine material in the past have led to conclusions in three categories: (1) intermixed nano-domains of two phases that are trigonal LiMO_2_ (where M represents all the transition metals present in the compound) and monoclinic Li_2_MnO_3_ (refs 11, 14, 15, 16, 17, 18), (2) a single monoclinic phase in the entire sample^19^,^20^,^21^ and (3) a single trigonal phase or a ‘solid solution' with the presence of a superstructure^19^,^22^. Bulk techniques such as powder X-ray or neutron diffraction are traditionally used for phase identification. However, the experimental neutron and X-ray diffraction patterns of LMRTMOs can be fitted to all three proposed models^19^,^23^. Conversely, methods such as aberration-corrected scanning transmission electron microscopy (STEM) provide high spatial resolution and have been used to suggest a single-phase nature of the oxides (for instance by Jarvis *et al.*^20^,^21^), but the results were to our knowledge always derived from small fields of view and therefore cannot exclude the possibility that other phases were present within the primary particles. It is unclear whether the report of three types of structure is a result of different synthesis conditions and/or compositions used by the various research groups, both of which have been shown to heavily influence the crystal structure of the oxides.

Furthermore, structural changes on the surface layers of LMRTMO were recently attributed to the effects of cycling^24^. Such transition metal-rich surface layers, however, have also been observed in pristine materials as reported in recent studies on Li_1.2_Ni_0.2_Mn_0.6_O_2_ (ref. 17) and Li_1.2_Ni_0.175_Mn_0.525_Co_0.1_O_2_ (ref. 25).

Thus, a better understanding of structural changes and phase stability of the cathodes demands comprehensive studies that unfold both the bulk structure and surface composition in LMRTMO. In this paper, we report structural and spectroscopic analyses performed on a variety of LMRTMO samples, including two crystal samples with discrete, well-formed primary particles and two commercial samples from different sources, by using complementary capabilities offered by aberration-corrected STEM, electron energy loss spectroscopy (EELS) and X-ray energy-dispersive spectroscopy (XEDS). We present unambiguous evidence that LMRTMOs are composed of a monoclinic single phase with three different variants in the bulk and a Co- and/or Ni-enriched spinel surface layer that is crystallographic facet dependent. Our study is mainly based on primary particles prepared using a molten-salt method with a composition of Li_1.2_(Ni_0.13_Mn_0.54_Co_0.13_)O_2_ having needle and plate morphologies as shown in Supplementary Fig. 1. This synthesis technique utilizes a liquid reaction medium to enable atomic-level mixing of the reactants and to allow for a homogeneous nucleation and growth of crystals from the flux at a relatively low temperature. Synthesis using this technique results in discrete primary particles that are impurity-free, making them ideal candidates for performing fundamental characterization using advanced high-spatial resolution techniques such as aberration-corrected STEM. For comparison, we also studied two commercial samples: TODA HE5050 having a composition of Li_1.2_Mn_0.55_Ni_0.15_Co_0.1_O_2_ and HCMR XLE2, which has a proprietary composition with a general formula Li_*y*_MO_2_, where *y*>1, M=Mn, Co and Ni and Mn>Ni>Co. For all the samples, we obtained three-dimensional structural information by imaging them using multiple zone axes, thus eliminating the ambiguities that can arise from the fact that materials with different structures can give similar two-dimensional projections. It should be emphasized that the present study is based on results obtained from LMRTMO that consists of Ni, Mn and Co as the transition metals and have relatively high amount of lithium. The species in the transition metal oxides (for example, elements other than Ni, Mn and Co) and the amount of lithium and/or manganese (for example, in case of LMRTMO with lithium content closer to stoichiometric LiMO_2_) can potentially have an effect on the structure of LMRTMO.

## Results

### Observation of monoclinic domains using STEM imaging

**Figure 1:**
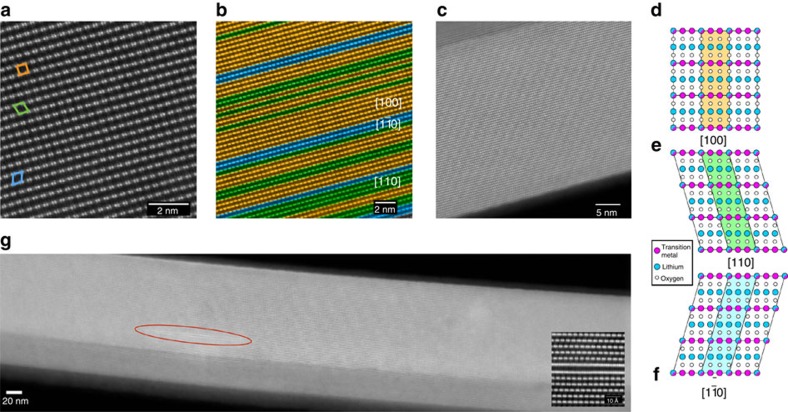
HAADF STEM images taken from the needle sample. (**a**) HAADF image showing the structure of LMRTMO needles. (**b**) Colour-coded HAADF image showing the variants of the monoclinic phase. (**c**) HAADF image with a larger field of view showing that domains of a single-phase, monoclinic structure are present throughout the primary particle. (**d**–**f**) Model showing the monoclinic structure in [110], [1
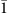
0] and [110] directions, respectively. (**g**) HAADF image showing that the structure is observed over the entire primary particle. Occasionally, transition metal-rich defects are observed as shown in the inset, which corresponds to the defect shown in the red oval.

To determine whether the primary particles consist of single phase or multiple phases using atomic-resolution images, it is necessary to check whether the projections that are unique to the respective structure are continuous throughout the primary particle. Figure 1a shows a high-angle annular dark field (HAADF) image that consists of bright columns corresponding to the transition metals. As seen in the micrograph, projections corresponding to [100], [1
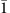
0] and [110] directions of the monoclinic phase, shown in the structural models in Fig. 1d–f, are found in the same field of view with the flips between the domains occurring on the (001) plane. It should be noted that HAADF images in this zone axis consist of doublets of transition metal columns. This projection of transition metals is unique to the monoclinic phase and impossible for a crystal consisting of a trigonal LiMO_2_ phase to produce in any orientation. Domains consisting of these three variants of the monoclinic phase are shown in orange, blue and green colours in a lower-magnification image in Fig. 1b. HAADF images taken in this direction have been reported in recent publications^26^,^27^,^28^, albeit with small fields of view covering only a small portion of the primary particles. In this study, we were able to take atomic-resolution images showing this structure throughout the primary particles, as shown in Fig. 1g taken from the needle-shaped sample, thus eliminating any doubts of presence of other phases in the primary particle. This was particularly obvious in the needle sample, where due to the small thickness of the particle, we were able to image the entire width of the primary particle in atomic resolution and images along the entire length of the particle unambiguously proved that although the particle is made of domains corresponding to the monoclinic variants, it entirely consists of a single phase. It should be noted that both the size and stacking of these domains in the direction normal to the transition metal layer have no apparent order, and the lattice cannot be translated along the *z*-direction although it can be translated along *x*- and *y*-directions. It can thus be said that these LMRTMOs have two-dimensional periodicity. The material is crystalline, but primary particles are not single crystals. Generally speaking, these materials can therefore be referred to as aperiodic crystals according to the modern definition of crystals^29^,^30^. It can also be seen from Fig. 1b that the domains usually flip between [100]_M_ and [110]_M_ or [1
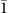
0]_M_, where the subscript M refers to the monoclinic unit cell, although occasionally reflection twins (flips between [110] and [1
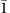
0]) were also observed, consistent with observations made by Jarvis *et al.*^27^. Similar observations were also made on the commercial HCMR XLE2 and TODA HE5050 materials as shown in Supplementary Figs 2b and 3b, respectively, where the variants of the monoclinic phase were found throughout the primary particles, except for some infrequently observed areas consisting of defects.

**Figure 2:**
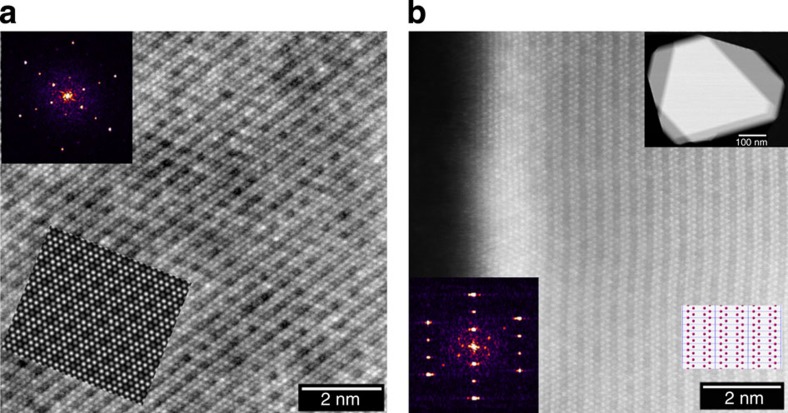
Projection of LMRTMO in [103] direction. (**a**) HAADF image taken from the needle sample showing all three variants, although only two of them are the prominently present as shown in the Hanning-masked FFT image. The inset also shows a STEM image simulated by multislice method under similar conditions (**b**) HAADF image taken from the plate sample where only one variant was prominent as also evident from the Hanning-masked FFT. Model showing projection of transition metals in the monoclinic structure in [103] direction is also shown in the inset.

Further studies on the bulk structure of the materials were performed by observing the samples in [103]_M_ (normal to the shared (001)_M_ plane) and the [001]_M_ directions. Figure 2a shows a HAADF image from the needle sample tilted to [103]_M_ zone axis. The criss-cross pattern of rows of transition metal columns corresponds to the presence of three projections of [103]_M_ (as shown in the lower right inset in Fig. 2b), although two of them are more prominently present in this example, as also evident from the diffractogram shown in the inset of Fig. 2a. Several particles, both from needles and plates, exhibited areas where only one variant was prominent, as demonstrated in Fig. 2b, that was taken from the plate sample. Similar results were also observed in the commercial TODA HE5050 sample, as shown in Supplementary Fig. 3c. It is interesting to note that in a recent study on TODA HE5050 material, Mohanty *et al.*^23^ concluded that this material was more likely a composite of trigonal and monoclinic phases based on results from temperature-based magnetic susceptibility measurements, which is contradictory to the conclusions from this study that clearly shows the absence of a composite structure based on direct observation using various zone axes.

**Figure 3:**
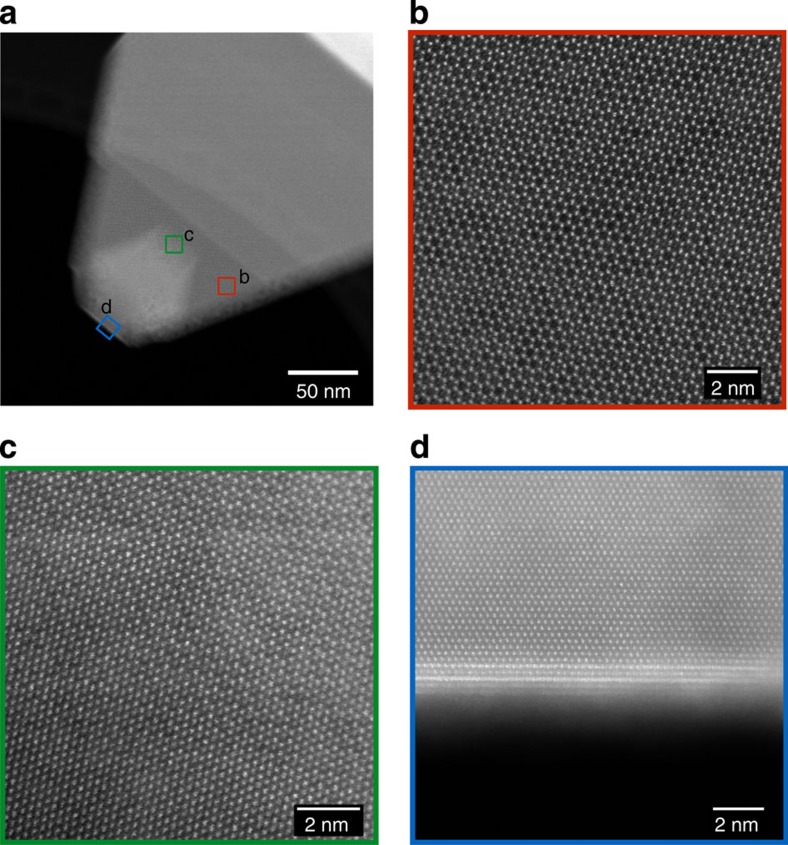
HAADF images taken in [001]_M_ zone axis. (**a**) a low-magnification image showing a terraced plate. (**b**) Image showing a honeycomb structure similar to monoclinic Li_2_MnO_3_ with predominantly a single variant. (**c**) Image from an area corresponding to different levels of stacking. Note the honeycomb-like structure and hexagonal-like structure in the top-right and bottom-left regions of the micrograph, respectively. (**d**) Image taken from the thick region shown in the bottom-left corner of **a**, indicating a region consisting of all three regions and showing a different structure on the surface.

While certain directions as shown in the above two examples uniquely show the presence of variants of monoclinic phase in the bulk, images taken in some directions can be quite misleading, which we believe has led to some of the contradicting reports in the literature. Figure 3 shows an interesting example of a plate that provided areas with the three variants in varying amounts due to the presence of terraces on them, as shown in the lower-magnification image in Fig. 3a. When a single variant is imaged in the [001]_M_ direction, a honeycomb or graphene-like projection is observed. Figure 3b shows a HAADF image from an area that predominantly consisted of a single variant. Closer inspection of the image shows that the centre of the hexagon exhibits some intensity, which is due to the fact that the column has shared Li and TM occupancy. In some areas, this intensity is higher. This higher intensity is not due to higher concentration of transition metals in lithium columns, but simply due to the effect of stacking of the variants as shown in Fig. 3c, where a honeycomb-like structure is observed on the top-right corner (corresponding to the region with a predominantly single variant) of the image, while a hexagonal-like image is observed in the bottom-left corner (corresponding to a thicker area where two or three variants are present). Thus, if a small area with two or three variants is observed in projection, as shown in the Fig. 3d corresponding to the thick area, it is possible to mistakenly assign them to the trigonal phase. Note that Figs 2a and 3d show the presence of a different phase on the surface that is discussed later in the paper.

### Characterization using EELS and XEDS

**Figure 4:**
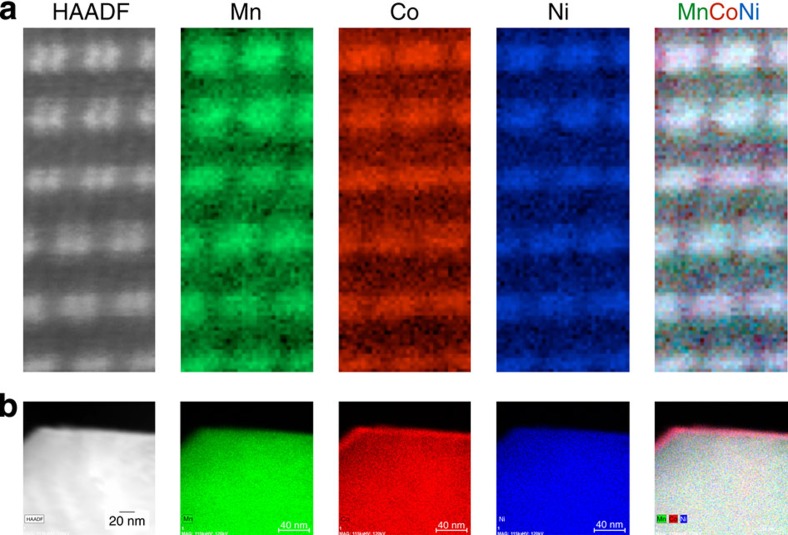
EELS and XEDS. (**a**) atomic-resolution EELS maps for the needle sample and (**b**) higher field-of-view XEDS maps taken on the plate sample showing uniform distribution of transition metals.

To confirm the presence of a single phase throughout the primary particle, and particularly the lack of Mn-rich domains as previously reported, we studied the elemental distribution of transition metals using both EELS and XEDS. Atomic-resolution EELS maps as shown in Fig. 4 were obtained on several regions in a primary particle, and the experiments were repeated on several particles from both needle and plate samples. Since the material is quite electron-beam sensitive, relatively low doses (<40 pA of current, which is lower than often used for EELS mapping but still high by comparison with true low-dose experiments^31^) of electrons were used and an improved signal-to-noise ratio was obtained using principal component analysis, which allowed the clear localization of transition metal atomic columns in the EELS maps^32^. Oxidation states of transition metals were calculated from the core-loss ionization edge intensities. L edges in transition metals result from excitations of 2p electrons into empty bound states and feature two white lines due to spin–orbit-split 2p_3/2_ and 2p_1/2_ levels to the available states in the 3d band. The ratio of intensity of these lines, called L_23_ ratio increases with decrease in oxidation states for 3d metals. By taking the L_3_/L_2_ ratio of the white lines (by applying a cross-section step function and then integrating the area under the peak corresponding to the L_3_ and L_2_ edges) as described by Pearson *et al.*^33^, the oxidation states of the elements present in our samples were found to be consistent with 4+, 3+ and 2+ for Mn, Co and Ni, respectively, as expected^33^. The EELS maps taken throughout the particle did not exhibit areas with relatively higher manganese concentration, which would be expected if the particle were a composite of Li_2_MnO_3_ and LiMO_2_ in the bulk of the particle. EELS maps taken using other zone axes also did not reveal any segregation of Mn in the bulk. The uniformity of transition metal distribution was confirmed by taking XEDS maps with large fields of view as shown in Fig. 4b, which was taken from a plate sample. Interestingly, the XEDS maps show that the surface layer on the edge of the particle, which imaging alone suggested may consist of a different crystallography than the bulk, exhibits clear cobalt segregation (with slightly higher than average Ni content). The structure and composition in this surface layer, as well as the oxidation states of transition metals therein, will now be discussed in detail.

### Analysis of the surface layer

**Figure 5:**
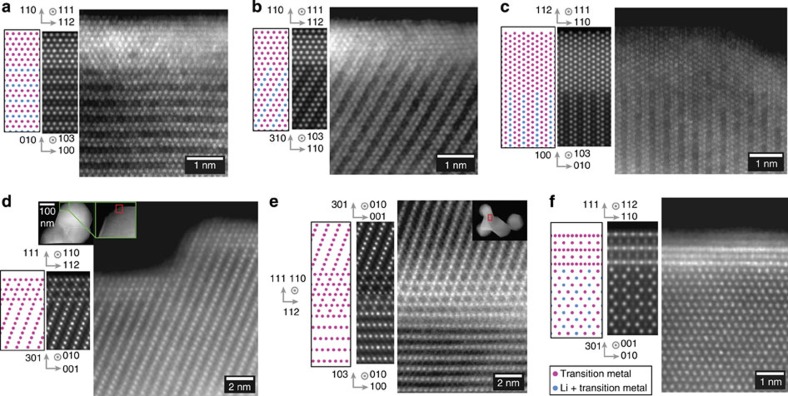
HAADF images alongside the STEM simulation image and model of the interface. (**a**) The surface layer is spinel viewed in [111]_S_ zone axis, while the bulk shows predominantly a single variant of the monoclinic structure. The interface is (010)_M_. (**b**,**c**) Spinel surface on other facets on the same particle, at 120 degrees from (010)_M_ as shown in **c** but also seen on facets normal to (010)_M_, as shown in the upper part of the image in **c**. (**d**,**e**) Spinel surface and the bulk in [110] and [010] zone axes, respectively. (**f**) Surface spinel in [112] zone axis and the bulk in the orientation equivalent to [001]_M_. Only one variant of the monoclinic structure is shown in the model and STEM simulation. HAADF images in **a**, **b**, **c** and **f** were recorded from the plate sample, while those in **d** and **e** were recorded from the commercial HCMR XLE2 sample.

Figure 5a shows a HAADF image of the plate sample in the orientation [001]_supercell_, equivalent to [103]_M_. In this plate, only one variant of the monoclinic structure is predominantly present, similar to the image shown in Fig. 2b, whereby the dark regions in the striped pattern correspond to rows of lithium columns, although some intensity from the presence of transition metals is observed due to the presence of other variant(s) in a small amount. In the surface layer, these rows of lower intensities are filled up, making a hexagonal pattern. This phase is typically ∼2 nm thick and is present on (010)_M_ facets, as shown in Fig. 5a, and at 120 degrees to (010)_M_ plane that corresponds to the other two variants of monoclinic (Fig. 5b), but is also seen on facets normal to (010)_M_ as shown on the lower part of the image in Fig. 5c. One possible interpretation, based on the intensities of these extra columns in the HAADF images, is that the lithium atoms are replaced by transition metal atoms and the intensities are due to the presence of antisite defects. However, after examining several particles in different orientations we were able to conclude that the surface layer had a different crystal structure, that of a spinel. In particular, the commercial HCMR XLE2 and TODA HE5050 materials have primary particles with an equiaxed morphology, which allowed us to view the primary particles in [010]_supercell_ or the equivalent [010]_M_ direction. HAADF images taken in this orientation, such as the one shown in Fig. 5d for the XLE2 material confirmed the presence of a spinel structure on selected surface facets of the particle. Figure 5d was used to deduce the orientation relationship between the bulk and the surface spinel, which is described below.


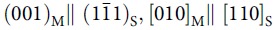


A model of the interface was prepared using this orientation relationship and it matched with experimental HAADF images taken with all other zone axes as shown in Fig. 5a–c,f. The zone axis for the spinel surface layer shown in Fig. 5a–c is [111]_S_, while that for Fig. 5f is [112]_S_. Interestingly, Fig. 5d–f shows that the crystallographic planes on which these surface layers terminate are {111}, which are known to be stable facets for spinels in general according to the Wulff theorem^34^,^35^.

**Figure 6:**
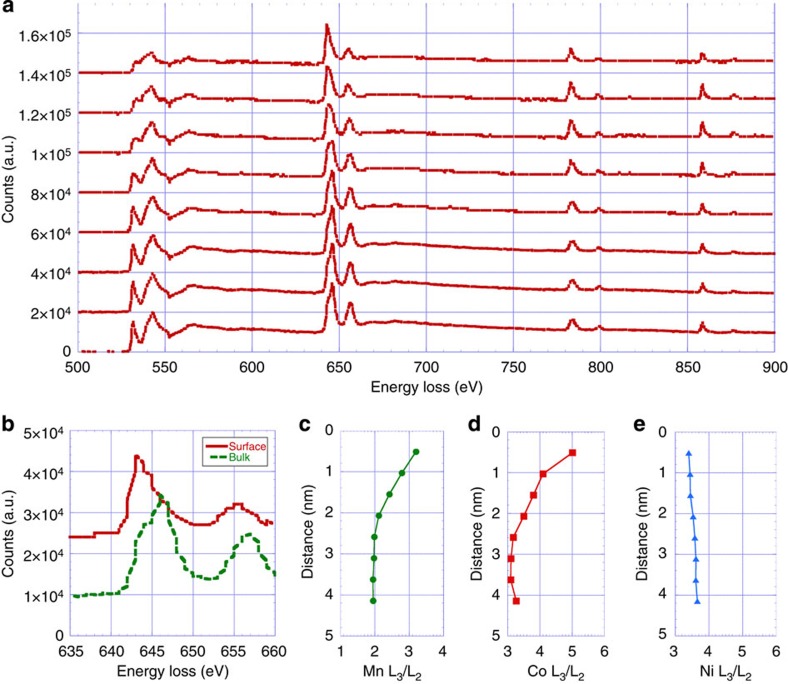
Analysis of EELS data. (**a**) series of EEL spectra showing the surface (top) and the bulk (bottom) corresponding to the HAADF image in Fig. 5a, (**b**) Mn L_3_ and L_3_ edges for the bulk and the surface, showing the presence of *e*_*g*_*−t*_*2g*_ split corresponding to the bulk region and the chemical shift to lower energy corresponding to the surface. (**c**,**d**,**e**) L_3_/L_2_ ratio corresponding to Mn, Co and Ni, respectively, showing decrease in oxidation states for Mn and Co at the surface.

EELS spectrum imaging revealed interesting changes that shed light on the structure of the bulk vis-à-vis that of the surface. These changes are observed via features in the spectra, such as the L_23_ ratio of transition metal white lines and the fine structure of O K and Mn L_2,3_ edges. Figure 6a shows a series of EEL spectra covering EELS edges from O to Ni, corresponding to surface and bulk regions of areas such as the one shown in Fig. 5a, where each spectrum was averaged from an area with three rows of atomic columns to improve signal-to-noise ratios. Figure 6c–e shows the L_23_ ratio for the transition metals. Although the exact valence is difficult to quantify precisely, the trends clearly show that the oxidation state of Mn and Co decreases as we approach the surface. The oxidation state of Ni stays constant at 2+, as expected.

Figure 6b shows an expanded view of the spectra close to the Mn edge, which shows that the Mn L_3_ edge corresponding to the surface is at a lower energy loss, compared with that of the bulk, indicating decrease in oxidation state at the surface. Moreover, the fine structure of the L_3_ shows a shoulder corresponding to the *e*_*g*_*−t*_*2g*_ split, which is associated with Mn^+4^ species with octahedral coordination^36^. The Mn L_3_ edge corresponding to the surface, on the other hand, does not clearly show this split as commonly observed in compounds with Mn in lower oxidation states. The overall signature of the bulk and the surface regions also corresponds to layered oxides and spinels, respectively. A higher oxidation state in the bulk is also indicated by the higher O pre-peak intensity observed in the bulk, compared with that in the surface, whose O signature resembles that of a spinel^37^. EELS spectrum imaging also revealed segregation of Co and Ni in the plates and needle samples on the surface. EELS spectrum imaging was also performed in the low-loss region, and analysis of the Li K-edge revealed that Li was present in these surfaces, although the concentration was lower than that observed in the bulk.

It should be noted that lithium-containing oxides, and specifically spinels are very susceptible to electron beam damage, and it has been recently shown that exposure to electron beam can also reduce the oxidation states of Mn and Co^38^,^39^. We also observed that the use of a higher dose or current led to the formation of antisite defects, with transition metals replacing Li atoms. This makes EELS experiments on such surfaces very challenging as also noted in a recent study on a similar LMRTMO material^25^. However, we were able to get reliable EELS data by performing spectrum imaging at 100 kV voltage with lower current (<40 pA) and short exposures and then improving the signal-to-noise ratio using principal component analysis^32^. Furthermore, as mentioned earlier, these surface layers were found only on certain crystallographic facets, and we confirmed that the change in oxidation state was not due to the effect of the electron beam by observing that the oxidation state of Mn and Co did not noticeably change when we scanned across surfaces that did not have these spinel domains using similar imaging conditions. Although the dose used was sufficient to provide us information on the decrease in oxidation states of transition metals and segregation of Co and Ni at the surface, we were not successful in localizing the atomic columns as we did in the case of the bulk regions (as shown in Fig. 4a), as the acquisition of such data sets required much higher dose rates (longer pixel dwell times) that in turn led to damage of the surface.

**Figure 7:**
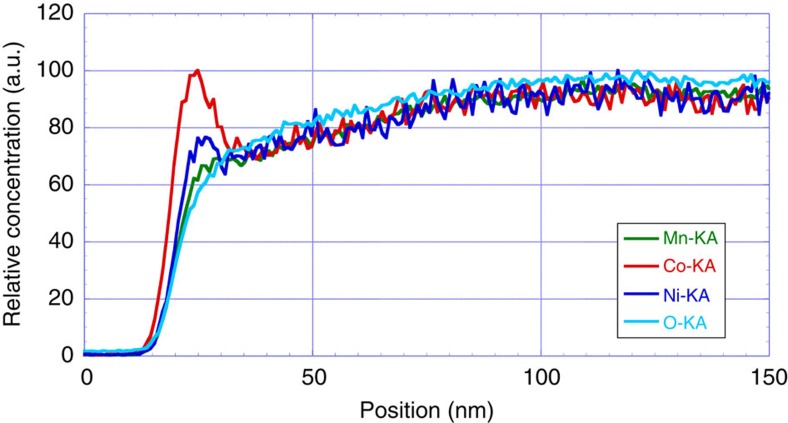
XEDS line scan across the spinel surface. These line scans corresponding to images in Fig. 4b for the plate sample show segregation of Co and Ni and depletion of O and Mn on the surface, relative to the bulk.

Further characterization of the spinel surface was achieved using XEDS mapping. Figure 7 shows an XEDS line scan across the surface shown in Fig. 4. Elemental information obtained from XEDS experiments confirmed that there was a lower concentration of oxygen at the surface, along with confirming the segregation of Co and Ni at the expense of Mn. It was also found that the relative concentration of Co and Ni on the surface depends on the original bulk composition. For example, the commercial HCMR XLE2 and TODA HE5050 materials, which had a higher Ni content exhibited higher concentration of Ni (compared with that of Co) on the surface, as shown in Supplementary Fig. 3.

**Figure 8:**
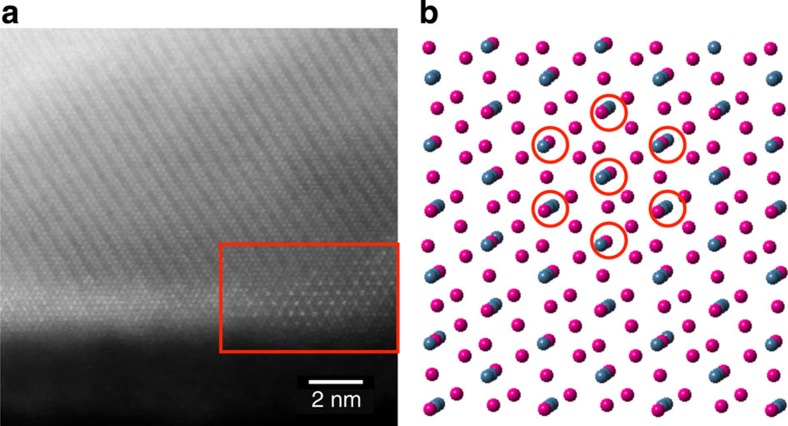
Antisite defects on the surface. (**a**) HAADF STEM image showing an apparently different structure in the thin areas (shown in the red-coloured rectangle). (**b**) A slightly tilted projection of spinel in [111] direction. The circled regions are the columns with mixed Li and TM atoms, which correspond to columns with antisite defects (TM in Li sites), giving higher intensity in HAADF images.

Finally, the HAADF STEM image in Fig. 8 taken from the plate sample shows the last piece of the puzzle, which represents certain areas (shown in the red rectangle) that showed an apparently different structure than that of a conventional spinel. As seen in the HAADF image, higher intensities were observed at distances twice that of adjacent columns, creating a ‘superstructure'. This was only observed occasionally on thin and tapered areas. We considered three possibilities that could potentially explain this anomaly. First, this could simply be the effect of thickness on the variation of intensities for different columns. STEM simulation indeed showed the effect of thickness as shown in Supplementary Fig. 5, but the intensities in the calculated HAADF images do not closely match those of the experimental images. Second, these antisite defects might also be formed due to exposure to the electron beam. We ruled out this possibility by observing such areas even when we rastered the beam to fresh areas by quickly moving the sample. This leaves us with the third possibility and the presence of antisite defects in the spinel structure, whereby some lithium sites are occupied by transition metals. This is also supported by results obtained by low-loss EELS, which showed a lower concentration of lithium at the surface. We can thus conclude that the pattern observed in these thin areas is due to a combination of the presence of antisite defects and the lower thickness. These antisite defects might also be present in the thicker regions such as the flat facet shown on the left side of Fig. 8a as evidenced by the non-uniform intensities in these regions, but the effect is less obvious due to the higher thickness in these regions. It should be noted that the antisite defects we are referring to indicate the presence of transition metals in the (tetrahedral) Li sites in the spinel structure found on the surface, and not to antisite defects found in the parent structure as proposed by Dixit *et al.*^25^ in a recent study on Li_1.2_Ni_0.175_Mn_0.525_Co_0.1_O_2_. The results from different microscopy and spectroscopy techniques are summarized in Table 1.

## Discussion

**Figure 9:**
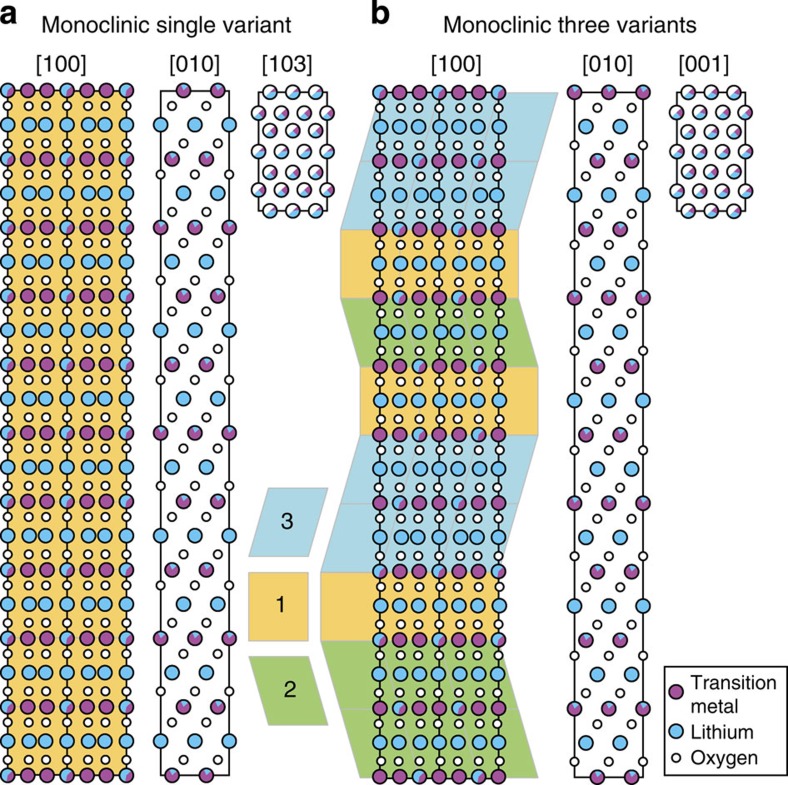
Structural model for LMRTMO. (**a**) LMRTMO with a single monoclinic variant and (**b**) three monoclinic variants in [100], [010] and [001] directions.

On the basis of the three-dimensional information obtained by HAADF images taken using various zone axes and supporting evidence from XEDS and EELS data, we can now conclude that the bulk of the primary particles is composed of domains that correspond to three different variants of monoclinic phase. Supercells generated by using a single variant of monoclinic unit cell and by randomizing monoclinic domains that are rotated 120 degrees and stacked on (001)_M_ planes are shown in Fig. 9a,b, respectively. The directions and their equivalence to a single crystal of the monoclinic structure are summarized in Supplementary Table 1. It is worth mentioning that the projection of the particle with three variants of the monoclinic structure in [010]_supercell_ direction and its equivalent directions (rotated 120 degrees along the *z*-direction) is indistinguishable from a trigonal structure, for example, in case of stoichiometric LiMO_2_ with 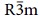 symmetry (when viewed in [010] or [100] directions) and also from a single crystal of the monoclinic phase. This is true not only for the projection of the transition metals as observed in HAADF images, but also for the projection of lighter oxygen and lithium atoms, as can be seen in annular bright-field images, which have been used to infer the presence of domains with trigonal structure in Li-rich-layered materials in recent studies^11^,^18^. For example, a projection similar to that shown in Supplementary Fig. 3d can also be obtained from a single crystal consisting of Li_2_MnO_3_ or LiNi_0.33_Mn_0.33_Co_0.33_O_2_ when observed in equivalent directions, and analysis using several zone axes is required to reveal the true structure of the material.

It has been shown that LMRTMOs convert to spinel^11^ or ‘spinel-like'^24^ upon cycling. More recently, it has been shown that surface layers with a different composition than that of the bulk are present even in pristine materials and are facet dependent^17^,^25^, which calls for careful consideration of facets and orientations of the particles when comparing pristine and cycled materials. The structure of this surface layer has not been solved in previous studies, to our knowledge. Gu *et al.* suggested that the surface layer in LiNi_0.5_Mn_0.5_O_2_ has the same structure as the bulk but with a different orientation, while more recently Dixit *et al.*^25^ suggested that the surface layer consisted of antisite defects with transition metals substituting on Li sites based on their study on Li_1.2_Ni_0.175_Mn_0.525_Co_0.1_O_2_. We believe that these discrepancies and ambiguities in the analysis of the surface layer structure arise due to the fact these conclusions are based on projections obtained from a single orientation and several different structures can give the same projections, especially in the [010]_M_ direction. To this end, we have observed the surface layer from different orientations to provide a complete, three-dimensional picture on the surface layer and its orientation relationship with the bulk structure. Using a multiple-technique approach, we deduced that the surface of Li-rich-layered oxides is a Co- and Ni-rich spinel with several antisite defects.

In conclusion, we have shown unambiguously by the use of electron probe-based techniques applied over a wide range of length scales that the bulk structure consists of variants of the monoclinic phase throughout the particles, except on the surface of certain facets, where ∼2-nm layers of spinel structure were found. The bulk structure was confirmed in four different samples with different morphologies and compositions, which indicates that this structure is commonly observed in this class of materials. This study not only solves the ambiguity in the structure determination of these materials but it also highlights the importance of atomic-resolution imaging and spectroscopy performed on multiple zone axes when carrying out such investigations. It should be emphasized that these results are relevant to LMRTMOs, and it will be interesting to see whether and how the bulk structure changes as the amount of Li and Mn is decreased and we plan to study this effect in the future. We hope that results from this study on pristine LMRTMOs will provide new directions to researchers studying the phase transformations in this class of materials and can be applied to potentially solve the problems such as voltage and capacity fade that are related to the surface and bulk structure. Indeed, the voltage profiles of initial charge/discharge of LMRTMOs have been so far conveniently divided into two regions where Li is removed from the two ‘components', namely, LiMO_2_ and Li_2_MnO_3_. The substantial proof on a single phase presented in this study calls for revisiting the interpretation of the voltage profiles. Furthermore, with a now clearer understanding of the surface structure, the facets where they are formed and their orientation relationship with the bulk, we can attempt to understand the effect of delithiation and cycling on different facets by using electron- and X-ray-based tomography techniques^40^,^41^,^42^,^43^ that can provide both chemical and morphological information in a wide length scale. Ultimately, such studies can guide us to engineer primary particles with tailored facets that would minimize the change of structure and hence the stress and strain in the cycled material.

## Methods

### Synthesis

Both needle and plate samples were prepared using the molten-salt synthesis method. Stoichiometric amounts of LiNO_3_, Mn(NO_3_)_2_·4H_2_O, Co(NO_3_)_2_·6H_2_O and Ni(NO_3_)_2_·6H_2_O and of the flux (KCl for needles and CsCl for plates) were mixed in a flux to a total transition metal molar ratio of 8 and 2, respectively. The mixture was dissolved in a small amount of water, which was removed by mild heating under agitation. The dried powder was transferred into an alumina crucible with a lid, heated at a rate of 3 °C min^−1^ and then soaked for 8 h at 800 °C for needles and 900 °C for plates before cooling to room temperature at a controlled cooling rate.

HCMR XLE2 material was obtained from Envia Systems. According to the manufacturer, it was prepared using a co-precipitation technique as described in patent number US 8,741,485. In this process, LMRTMO is prepared using a solid-state method, whereby a lithium source is added to a metal carbonate that is precipitated from an aqueous solution of metal salts and then calcined at 900 °C. TODA HE5050 material was obtained from TODA KOGYO Corp.

### Electron microscopy

The needles and plate samples were studied using a probe-corrected microscope operating at 100 kV accelerating voltage using a convergence angle of ∼30 mrad and a collection angle for HAADF images that was calibrated at 82–190 mrad. The commercial samples (Envia and Toda) were observed using a probe-corrected microscope operating at 80 kV accelerating voltage using a convergence angle of ∼30 mrad and a collection angle for HAADF images of 60–180 mrad, except for the images in Supplementary Fig. 3a,b,d, which were taken using the same microscope at 300 kV accelerating voltage, a convergence angle of ∼17 mrad and a collection angle of 68–340 mrad. XEDS maps with sub-nanometre resolution were obtained at 120 kV accelerating voltage using an FEI Titan microscope equipped with a quad-detector system. Samples for electron microscopy were prepared by drop casting a sonicated solution of LMRTMOs and anhydrous ethanol. HAADF STEM simulations were performed using a custom code that was prepared using details provided in ref. 44.

## Additional information

**How to cite this article:** Shukla, A. K. *et al.* Unravelling structural ambiguities in lithium- and manganese-rich transition metal oxides. *Nat. Commun.* 6:8711 doi: 10.1038/ncomms9711 (2015).

## Supplementary Material

Supplementary Information Supplementary Figures 1-5 and Supplementary Table 1

## Figures and Tables

**Table 1:** Summary of results.

**Technique**	**Conclusion**
HAADF STEM imaging	Spinel structure on the surface based on [010], [121] and [111] zone axes, some antisite defects in the spinel.
Core-loss EELS	O-edge signature and L_3_/L_2_ ratios indicate lower oxidation states of Mn and Co.
Li K edge/low-loss EELS	Li is present, but in lower concentration compared with the bulk. Li K edge indicates a spinel structure in the surface and layered in the bulk.
XEDS	Co and Ni segregation on the surface, Mn and O depletion.

EELS, electron energy loss spectroscopy; HAADF, high-angle annular dark field; STEM, scanning transmission electron microscopy; XEDS, X-ray energy-dispersive spectroscopy.
