# Supplementary material for: Unravelling structural ambiguities in lithium- and manganese-rich transition metal oxides
Source: Nat Commun. 2015 Oct 29;6:8711. doi: 10.1038/ncomms9711 (PMC4846316; doi:10.1038/ncomms9711)
Supplement: Supplementary Information — Supplementary Figures 1-5 and Supplementary Table 1 [file ncomms9711-s1.pdf]

Supplementary Figures

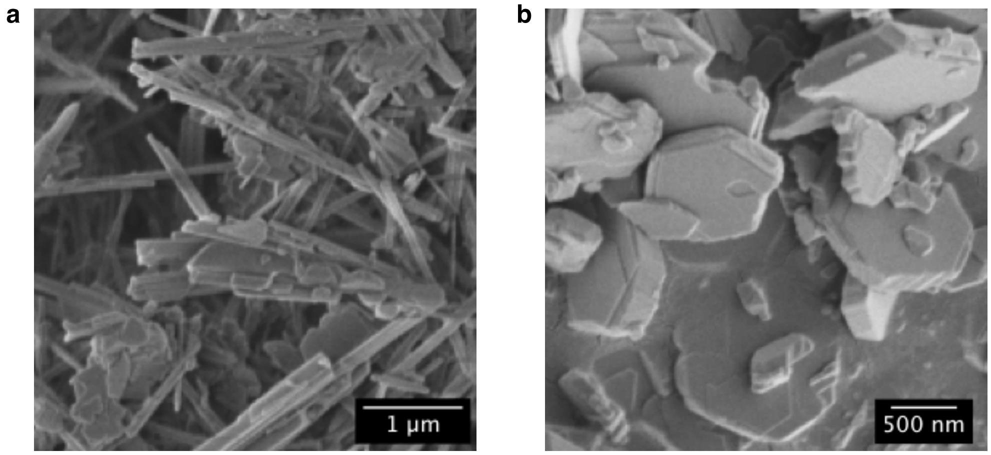

Supplementary Figure 1: SEM images for **a**, needles and **b**, plates showing the morphology of LRTMO primary particles.

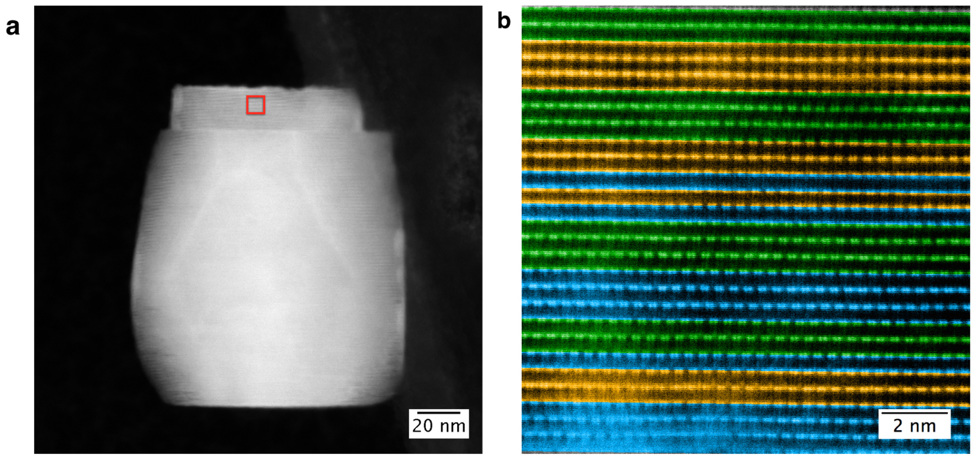

Supplementary Figure 2: HAADF STEM images showing the structure in commercial material HCMR^{TM}XLE2 **a,** Low magnification image showing a primary particle **b,** Higher magnification image using from the particle in **a** showing the domains representing monoclinic structure in $[1\,1\,0]$, $[1\,\bar{1}\,0]$ and $[1\,1\,0]$ directions.

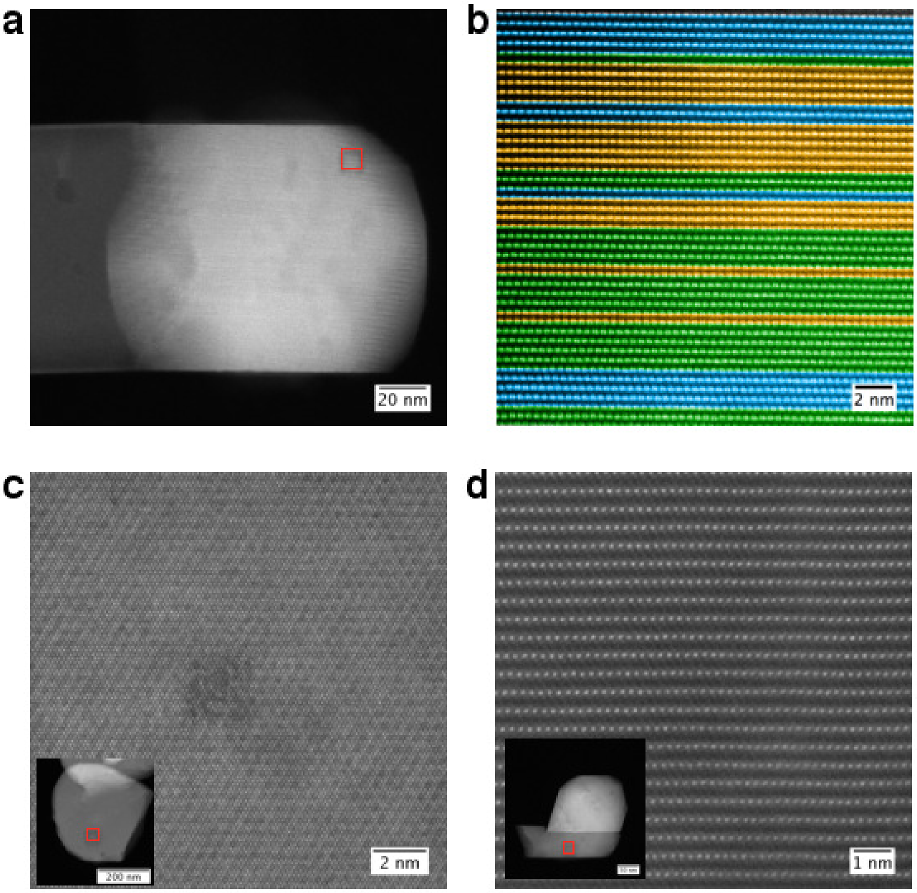

Supplementary Figure 3: HAADF STEM images showing the structure in commercial material Toda HE5050 **a,** Low magnification image taken using $[100]_{\text{supercell}}$ zone axis. **b,** image showing the domains representing monoclinic structure in $[1\,1\,0]$, $[1\,\bar{1}\,0]$ and $[1\,1\,0]$ directions, **c,** image showing the criss-cross pattern representing three variants of monoclinic phase in $[1\,0\,3]$ direction. **d,** image taken using $[010]_{\text{monoclinic}}$ zone axis.

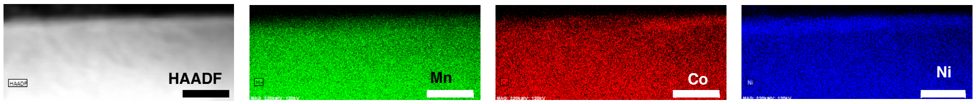

Supplementary Figure 4: XEDS maps taken on HCMR^{TM}XLE2 sample showing Ni segregation on the surface. The scale bar is 20 nm.

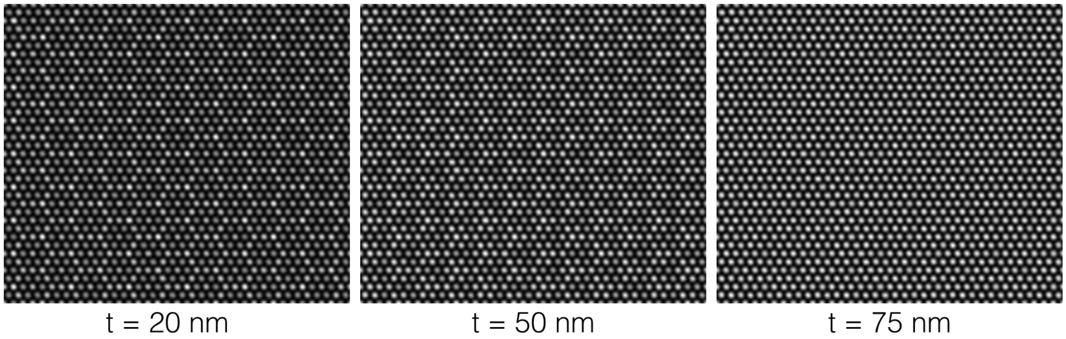

Supplementary Figure 5: STEM Simulation of spinel using different thicknesses.

Supplementary Table

Supplementary Table 1: Equivalent directions

**Monoclinic**	**Supercell**
[1 0 0], [1 1 0] and [1 $\bar{1}$ 0]	[1 0 0]
[0 1 0]	[0 1 0]
[1 0 3]	[0 0 1]
